# THE NATIONAL CONFERENCE WITH INTERNATIONAL PARTICIPATION

**Published:** 2017

The subject proposed entails an interdisciplinary theoretical research through the management applied in the sanatorium, a manner in which, by observing the Romanian laws in the matter of health, education, ecology, sustainable development, and socially, they were harmoniously and beneficially applied in our policies.

Given the definition of management, valid in any type of institution, as a complex process carried out on grounds of decisions adopted and materialized in activities to achieve some economic-social objectives, a trial has been made to answer how it could be implemented profitably on a long term, within a public unit subordinated to the Ministry of Health, by using the raw material of Techirghiol Lake as a basis and by applying the same conventional standards for an infinite number of patients and several hundred employees. 

To that end, medical management was aimed towards three directions: strategy of the institution, management of human resources and relation with patients. 

The medical management strategy of Techirghiol Balnear and Recovery Sanatorium started from the redesigning of the decisional system, based on the policies set in the management plan by means of which, two years ago, I was given the position of manager, upon contest. During these two years, the management team focused on improving the medical act as an alternative to the Romanian balnear tourism market, deepening the knowledge on the preferences of people by carrying out a professional survey. Thus, the social representation of the target audience was set. It was clear that the medium and elder patients, with severe health issues and average and small incomes, who used the balnear treatments and the virtues of Techirghiol Lake, in which they trusted unconditionally as a last hope of life, were dominant. The healing capacity of Techirghiol Lake was, nevertheless, renowned, starting from its legend, image brand to being the only form of non-aggressive and efficient form of treatment for a series of degenerative diseases.

However, a green management, which is a component of the general management system, also has to be applied, including responsibilities, practices, procedures, processes, and resources required to implement and maintain environmental policies. In addition, given this incommensurable wealth and gift of health, Techirghiol Lake, our strategies and decisions must also include the protection and the correct and rational exploitation of Techirghiol mud. So, the observation of the rich laws in the matter, which are not fully adapted to the European laws, as well as the orders, government decisions, norms, standards, ordering impact surveys, environmental analyses, risk assessments, monitoring programs, and compliance programs in cooperation with certified bodies, must be taken into account.

