# Supplementary material for: THE NATIONAL CONFERENCE WITH INTERNATIONAL PARTICIPATION
Source: J Med Life. 2017;10(Spec Iss 3):1–21. (PMC6088552)
Supplement: Related texts

# Meeting report: THE IMPACT OF THE THERAPY WITH NATURAL FACTORS ON THE QUALITY OF LIFE IN LUMBAR DISK HERNIA

**Authors:** EV Ionescu, N Calotă, MF Drăgoi, M Florea, R Badiu, G Lilios, LE Stanciu

**Affiliations:** * Techirghiol Balnear and Rehabilitation Sanatorium, Techirghiol, Constanţa, Romania; ** Faculty of Medicine, “Ovidius” University Constanţa, Constanța, Romania; *** ”Sf. Apostol Andrei” Clinical Emergency County Hospital, Constanţa, Romania

**Introduction.** Lumbar disk hernia is a frequent disease, worsened by repeated professional or sport efforts. Disk hernias can appear in a traumatic context, at 1-1,5%, but, most frequently, disk hernia is the last stage of degradation of a dehydrated, fissured disk, which disorganizes and eventually breaks. The management of disk hernia is complex and requires a multidisciplinary approach, with real chances only when neurosurgeons and rehabilitation/ balneologists are involved. Techirghiol benefits from special natural conditions, very good for health, including climate, sapropelic mud, salty lake water, bringing real benefits for lumbar vertebral disorders.

**Materials and methods.** 25 patients with lumbar disk hernia, who were hospitalized in the period June-July 2016, were followed for this study. The patients were selected based on a research protocol regarding clinical features and a functional evaluation file for each patient. The treatment was applied daily to the patients admitted to Techirghiol Balnear and Rehabilitation Sanatorium and consisted of balnear and adjuvant procedures (electrotherapy, kinesiotherapy, mesotherapy).

**Results.** The daily routine activities of patients were considerably improved after two weeks of complex balnear-physical treatment, with a significant progress of the patient’s quality of life.

**Conclusions.** The early implementation of a medical rehabilitation treatment produces an obviously better evolution than if only medical treatment had been applied.

# Meeting report: CLOSTRIDIUM DIFFICILE INFECTION – UPDATE 2017

**Authors:** AE Petcu, IM Dumitru, CS Cambrea, S Rugină

**Affiliations:** * Ovidius University, Constanța, Romania; ** Clinical Infectious Diseases Hospital, Constanța, Romania

**Argument.** Due to the increasing resistance of most bacteria to antibiotics in Romania, broad-spectrum antibiotics that have increased the risk of Clostridium difficile infection have been used. Also, the annual increase in the incidence of Clostridium difficile infection was correlated, according to studies, more frequently with hospitalization, surgery and immunodepression of various causes.

**Objectives.** Analyzing the number of cases hospitalized in a period of almost 5 years in terms of incidence, age, sex, and relapse.

**Methodology.** A retrospective study of 324 cases admitted to the Clinical Hospital of Infectious Diseases in Constanta, from January 2013 to August 2017, was performed.

**Results.** Of the 324 admissions, 36 were registered in 2013, rising to 73 in 2014, 82 in 2015, 73 in 2016, and 60 in 2017. The highest number of cases from a gender perspective was for female sex (60.12%). The age group with a higher incidence of cases was 50-69 years, with a total of 129 cases. The total number of episodes of relapse was 39, of which most of the patients had one episode (36 patients).

**Conclusions.** 1. The annual increase in the total number of hospitalized patients, however, with a decrease in the incidence of recurrent cases. 2. The highest number of cases was observed in patients aged 50-69 years, and in female patients.

**Keywords:** clostridium, hospitalized, relapse

# Meeting report: THE IMPORTANCE OF EVALUATING 25-HYDROXY VITAMIN D IN ANKYLOSING SPONDYLITIS

**Authors:** G Lilios, EV Ionescu, EO Lilios

**Affiliations:** * ”Ovidius” University, Constanta, Romania; ** *”Carol Davila” University of Medicine and Pharmacy Bucharest, Romania

Ankylosing spondylitis (AS) is a chronic inflammatory disease that primarily affects the axial skeleton.

The purpose of this study was to highlight the evolution in dynamics of the biochemical parameters (as markers of inflammation and evaluation of vitamin D levels) in patients of the test group diagnosed with AS. The test group consisted of 22 male HLA-B27 positive subjects.

The results of this study indicated statistically significant decreased levels of serum 25-hydroxy vitamin D, pT (α < 0,05) in the test group subjects compared to the control group (20 clinically healthy subjects), which indirectly confirmed the severity of bone tissue damage, through metabolic dysfunction, chronic inflammatory syndrome, increased bone resorption and favoring spinal cord deformation.

This study revealed connections between serum TNFα, the humoral inflammatory activity and the biochemical markers of bone metabolism, alongside the clinical evolution of the patients with AS. It is known that vitamin D deficiency present in AS is induced by pathophysiological mechanisms related to the marked growth of TNFα, which disrupts the renal activity of 24-hydroxylase, by reducing its production rate. Studies revealed a vitamin D deficiency in patients with AS, as an indicator of bone resorption, being a valuable marker of detecting the loss of bone matter in AS.

The catabolic state in which serum TNFα levels are increased may play an important role in osteoporosis pathogeny in AS. Although in the context of the disease, osteoporosis induces bone formation and bridges with invalidating implications (spinal cord and sacroiliac joints ankylosis, spinal cord deformations), osteoporosis by bone resorption is a well known complication of AS.

Taking into account that osteoporosis is a systemic affection responsible for bone mass loss, it is very important to understand the etiopathogenic and physiopathological mechanisms that lead to the early induction of osteoporosis in order to offer an appropriate advice and suggest the daily administration of vitamin D and calcium for preventive or therapeutic purposes.

**Keywords:** ankylosing spondylitis, 25-hydroxy vitamin D, serum TNFα, osteoporosis

# Meeting report: MAXIMIZATION OF boDMARDs AND csDMARDs THERAPY EFFECTS IN REUMATOID ARTHRITIS BY PHYSICAL-KINETIC THERAPY

**Authors:** IC Mihailov, L Peniu

**Affiliations:** * Department of Medical Semiology, Faculty of Medicine, “Ovidius” University, Constanța, Romania; Clinical Port Hospital of Constanța, Constanța, Romania; IInd Medical Department, Constanța, Romania

**Introduction.** The aim of this study was to obtain information on the effect of kinesiotherapy and physiotherapy in patients with rheumatoid arthritis in boDMARDS and csDMARDS patients in clinical inactivity (disease activity score (DAS)28 less than 2.6) or low activity (DAS28 between 2.6- 3.2).

**Objective.** The objective of the study was to highlight an improvement in the indices of disease activity after 10 days of physiotherapy and kinesiotherapy.

**Materials and methods.** Two measurements were performed: at the beginning of the study and 10 days after the physical-kinetic treatment, followed by a third measurement 3 months after the treatment. The disease activity indices compared were the following: DAS28, erythrocyte sedimentation rate (ESR), C reactive protein (CRP), visual analogue scale, painful joints, swollen joints, clinical disease activity index (CDAI), simplified disease activity index (SDAI), isometric contraction that determines flexion and knee extension in patients with rheumatoid arthritis and these parameters were observed in dynamics after performing the physical-kinetic treatments. The group included 27 patients out of whom: 19 patients were treated with csDMARDs (84% methotrexate) (11 clinical remission and 8 low-activity patients) and 8 patients treated with boDMARDs (89% rituximab) (5 patients in clinical remission and 3 patients with low disease activity). Patients were tested on day 1 of the study, followed by a physical-kinetic treatment until the end of the 10 days when a new test was performed. The physical-kinetic treatment consisted of the following: four-cell galvanic baths, paraffin cataplasm, ultrasound, TENS and kinesiotherapy. Subsequently, by the end of the 3 months, patients underwent an adapted physical therapy program at home under supervision and telephone guidance.

**Results.** A health assessment questionnaire (HAQ) had the average value of 0.98 before the balnear-physical treatment and 0.75 after the treatment, showing a significant improvement (p < 0.05) and both disease activity indexes (DAS28, CRP and DAS28, ESR, SDAI and CDAI), as measured by device (Torque index, real force increased, force measured with lever), decreased significantly (p < 0.05).

**Conclusions.** The physical and kinetic treatment, in conjunction with csDMARDs and boDMARDs, can lead to a significant decrease in the disease activity indexes and may maintain the remission of the disease or may result in low activity scores in remission. It is important that while inactive, patients are prevented twice-yearly by physical-kinetic treatment, as there is a clear improvement in the quality of life.

**Keywords:** physiotherapy, activity score, treatment, remission

# Meeting report: PSEUDARTHROSIS OF THE PATELLA

**Authors:** S Demirgian, I Lascu, A Lulea

**Introduction.** Pseudarthrosis is the complete absence of a fracture consolidation within 1-2 months over the usual consolidation time. In pseudarthrosis, the bone callus is not formed or is defective, which creates pseudoarticulation between the two fractured bone fragments that are more or less mobile from each other; meaning laxa pseudarthrosis or tight pseudarthrosis.

**Objectives.** The presentation analyzed the side effects of a combined fracture operated in association with a depressive syndrome.

**Material and method.** The case of a 60-year-old woman, who presented to our clinic for pain and function laesa of the right knee, was presented. The patient was evaluated anamnestically, clinically and paraclinical. Visual Analog Scale was used as an assessment tool.

**Results and discussions.** Because the orthopedic pathology was associated with a psychiatric disorder, we were able to state that the postoperative and medical recovery evolution was unfavorable, with the occurrence of patellar pseudarthrosis. Despite this, the patient presented a range of motion close to the normal limits, but with a subjective assessment of pain close to the upper limits.

**Conclusions.** The particularity of the case was due to the low incidence of patellar pseudarthrosis, the association of depressive syndrome, and the fact that the patient required five surgical interventions to support multiple iterative fractures.

**Keywords:** pseudarthrosis, comminuted fracture, patella, depressive syndrome, pain and function laesa

# Meeting report: TECHIRGHIOL BALNEAR AND REHABILITATION SANATORIUM EXCELLENCE IN MEDICAL RECOVERY

**Authors:** LE Stanciu, EV Ionescu, V Marin, MF Drăgoi

**Affiliations:** * Techirghiol Balnear and Rehabilitation Sanatorium, Constanța, Romania; ** Faculty of Medicine, “Ovidius” University, Constanța, Romania; *** “Sf. Apostol Andrei” County Emergency Clinical Hospital, Constanta, Romania

Techirghiol Balnear and Rehabilitation Sanatorium, an elite unit providing medical treatment using balnear therapeutic factors, was ranked first in the top of the spa resorts in Romania for the second consecutive year. The Romanian Balneology Association performs this ranking annually. This prize honors and at the same time obliges it to provide exceptional healthcare services that are competitive with international medical services.

Diversified pathologies are treated in this medical unit, the hospitalized patients being both Romanian and foreign. An important feature of admissions is that patients regularly return to treatment, finding permanent improvements in healthcare provision. Within the medical base, treatments are performed by using modern medical equipment, the medical personnel being permanently present at national and international medical congresses, to align with standards, with new therapies that provide modern, personalized medical treatments, tailored to all the patients who address this medical unit.

Currently, within the kinesiotherapy department, Walker View treadmill performs tests for walking recovery and motion analysis with dynamic goniometry.

The research part is well represented in the sanatorium, which is constantly conducting scientific studies on the natural therapeutic factors used, in order to identify other possible medical indications of treatment with sapropelic mud in the Techirghiol area.

**Keywords:** sapropelic mud, spa resort, Techirghiol

# Meeting report: THE EVOLUTION OF THE PHYSICAL AND CHEMICAL PROPERTIES OF TECHIRGHIOL LAKE FROM 1975 TO 2015

**Authors:** G Gheorghievici, M Cosmoiu, M Mustaţă, I Tănase

**Affiliations:** * National Institute of Rehabilitation, Physical Medicine and Balneoclimatology, Bucharest, Romania

The study presents the evolution of Techirghiol Lake on a period of 4 decades, evidencing the water and its mud that represent unique therapeutic elements in an international context, their efficiency manifesting in the social and economic field, especially regarding the healthcare aspects. 

The paper presents the water balance, generally deficient in the semiarid climate conditions from the South of Dobrogea, due to which the lake has developed a high mineralization level in time. There have been periods with high or diminished levels of water in the evolution of the lake, associated with an increase and decrease of mineralization, reflecting the alternation of the rainy periods with the dryness ones. During this interval, there have been periods sufficiently long of an increased level of water, under the pressure of the supply of sweet water gained by the lake, which endangered the natural process of formation of the therapeutic mud. 

The study evidenced the peloidogenesis process as a complex of phenomena conditioned by the specific of the formation zone, the climate characteristic, and the physical, chemical, biological, and microbiological properties that react promptly to the modification of any environmental parameter. In the case of Techirghiol Lake, the modification of the water regime through the supply of sweet water was triggered by chain processes for the other links. 

The main influence for the quality and quantity of the mud which is annually formed in the lake are those observed at the level of the environmental physical and chemical factors, the nature and the distribution specificity of the mud producer (flora, fauna), of the consumers and decomposers (bacteria, fungi). 

The Chemistry and Microbiology Laboratory of the National Institute of Rehabilitation, Physical Medicine, and Balneoclimatology performed a series of researches and periodic observations regarding the Techirghiol ecosystem, starting from the premise that the therapeutic character of the existing mud and the one that annually forms must be maintained. In addition, the constant consequences were presented from a physical and chemical point of view between 1975 and 2015, because of the of the water lake evacuation through sea pumping at the beginning of the 70’.

# Meeting report: MANAGEMENT OF NEUROGENIC BLADDER IN PATIENTS WITH SCI

**Authors:** I Petrusca, D Cinteza, O Naglabeala

The neurogenic bladder is defined as a dysfunctional bladder due to neurogenic causes and it is based on a detrusor hyperactivity often combined with detrusor-intern sphincter dyssynergia. Both storing and bladder emptying functions are affected.

The main symptom in spinal injuries is urge incontinence. Bladder empties too often and quickly, similar to brain injuries, but the external sphincter becomes spastic and shows paradoxical contractions. If the bladder and external sphincter become spastic at the same time, the individual feels an urgent needed to urinate but can only eliminate very small amounts of urine.

The initial phase consists of the following: spinal shock, flaccid paralysis, reduced or absent somatic reflex activity, absent anal and bulbocavernosus reflexes, decreased autonomic system activity. It typically lasts for 6-12 weeks and is often prolonged.

After the spinal shock phase, the detrusor activity resumes, but becomes hyperactive and the detrusor hyperreflexia is established. Depending on the lesion level, DSD-DH can be installed.

Conservative treatment is the first line treatment (catheterization/ drug treatment/ behavioral therapy/ KT/ ET) and its purpose is to prevent complications and increase the patient’s quality of life.

Urodynamic evaluation is required for a complete diagnosis of neurogenic dysfunction.

The objectives of the rehabilitation treatment are the following: patient education, increased control over the phases of urination, relief of symptoms, prevention/ treatment of complications, preservation of the kidney function, improvement of the quality of life.

# Meeting report: THE IMPACT OF ORGANIC MATERIAL SOURCES TYPOLOGY EXISTENT IN TECHIRGHIOL PELOIDOGEN ECOSYSTEM ON THE PHYSICAL AND CHEMICAL CHARACTERISTICS OF THE PREFORMED MUD

**Authors:** L Gheorghievici, M Cosmoiu, I Pompei, M Mustaţă, I Tănase

**Affiliations:** * National Institute of Rehabilitation, Physical Medicine, and Balneoclimatology, Bucharest, Romania

Techirghiol Lake is an ecosystem that maintains its peloidogenesis potential, although its capacity of generating mud with therapeutic properties continually varies, being determined by the permanent modification of the abiotic factors that influence the biocenosis, and accordingly the composition of the plankton and the development of the aquatic vegetation. 

The microalgae represent the basis of the primary productivity in the saline lacustric ecosystems. This type of biocenosis includes a low amount of species but a large number of individuals. The microalgae are implicated in the purification of the water, the formation of mud, and the insurance of oxygen necessary for all aerobe organisms. 

The study proposes the evaluation of the interrelation between the type of sources of organic material existent in the peloidogenesis ecosystems and the chemical composition particularities of the neoformed mud. The stability of the modality in which the structure modifications of the microalgae communities, induced by the variation of the abiotic factors, determines the formation of a sediment with specific chemical properties represented the objective of our study. 

The biocenosis of Techirghiol Lake tends to position itself in a stable equilibrium with the environmental factors; three necessary and sufficient elements for the formation of a mud with therapeutic properties are developed in this lake: organic material+, specific microbiota+, a certain quantity of mineral salts. 

The primary producers of Techirghiol ecosystem are the microalgae and the macrophytic algae Cladophora crystallina. They presently develop in proportions that ensure a sufficient supply or organic material to the peloidogenesis process.

The specific microbiota from the sediment includes types of microorganisms and densities proportionally adapted to the source of organic material existent in the ecosystem; through their activity of decomposition, they supply the final products that will shape the chemical structure profile characteristic for the mud from Techirghiol.

In order to maintain the peloidogenesis character of Techirghiol Lake and the formation of mud with therapeutic properties in sufficient quantities, the protection of the ecosystem and its medium is needed together with the long term monitoring of the pollution factors, biotic and abiotic, that can interfere and unbalance the ecosystem.

# Meeting report: CONSTRAINT-INDUCED MOVEMENT THERAPHY AFTER STROKE

**Authors:** L Cioc, D Cinteza, I Petrusca, SI Neagoie, RIA Popovici

**Introduction.** Stroke is a major problem, because patients who survive often remain with severe motor disabilities that compromise their quality of life. Approximately 80% of the stroke survivors have upper limb paresis. The disability of the upper limb has implications for the management of daily life activities (ADL). Constraint induced movement therapy is a commonly used method for the improvement of functionality of the upper limb after stroke.

**Objectives.** The goal of this paper was to present and explain the essential elements of the protocol of constraint therapy in patients, after stroke, and also to determine the efficiency of therapy. 

**Methods.** Information from international clinical trials, treatment guidelines, and practical experience of the clinical data on indications, patient selection, and efficiency of therapies.

**Results.** Initially, it was developed as a method for chronic stages in patients with paretic deficiency. Several forms of modified constraint therapy have been developed until present, resulting into heterogeneity in terms of content, application time, and intensity of therapy. Clinical trials related to mCIMT failed to provide a detailed description of the protocol used. A recent meta-analysis of these studies emphasized the positive effects of mCIMT in the first few weeks and suggested that the modified form (with a shorter duration of repetitive exercise of less than 3 hours a day) is more beneficial. 

**Conclusions.** The goal of therapy is to increase the functionality of the upper arm in the first weeks after stroke. The early onset of therapy is based on increased neuroplasticity that occurs in the first 3-4 weeks. The protocol uses a gradual approach that aims to increase functionality by combating compensation strategies. The essential elements of the protocol are the constraint element and the repetitive training.

# Meeting report: THE SULFUR IN THE MINERAL WATER

**Authors:** M Cosmoiu, L Gheorghievici, I Pompei, Gh Gheorghievici, I Tănase, M Mustaţă, Ș Bujoaica, M Florea

**Affiliations:** * Therapeutical Natural Factors Department, National Institute of Physical Medicine & Rehabilitation, Bucharest, Romania

The sulphurous mineral waters represent complex solutions of electrolytes in which sulfur compounds in state of oxidation 2 exist, i.e. H2S, HS-, and more rarely S2-, which gives them specific characters reflected in the therapeutic effect.

The large possibilities of valorization in the medical field needed the attention on the physical-chemical composition and processes of this kind in the past and are also requested nowadays, with their direct reflection in the capacity of keeping the therapeutic characters.

The analytical data regarding the sulphurous waters accumulated over the years have emphasized a great variety of physical-chemical compounds concerning both the quality and the quantity. They have also emphasized the variations of the redox potential in time on some types of sulphurous waters as indicators of some characteristic processes of these solutions.

The purpose of the present work itself was the notification of the physical-chemical balances mobility of the sulphurous mineral waters in time, of the generating causes, as well as the determination of some correspondences between the physical-chemical indicators alteration and the therapeutical effects. 

The premise from which the interpretation of these physical-chemical elements of these mineral waters started was that they represent electrolytes solutions of a determined ionic strength, which have an evolution in time and are strongly influenced by the interactions between the solution ions. The size of the ionic strength influences the activity coefficients of the existing ions and by these, the processes in which the ions participate in solution.

The pH is a value both dependant on the solution ionic strength and the temperature, a fact which should be considered in the values estimation. Its mobility in time, in the sulphurous mineral waters is an indicator of the processes that take place in them, such as ionization phenomena, like the one in which H2S takes part:

• salts hydrolyze phenomenon

• precipitation phenomenon (ex. the insolvable sulfurs formation)

• oxidation-reduction phenomenon, such as the one with the participation of the sulphur compounds in different states of oxidation.

The behavior of the sulphurous waters in time depends on a series of physical–chemical factors, among which the following can be mentioned:

1. the temperature in emergency cases and its variation in time

2. the chemical composition and the ionic strength of the solution respectively

3. the concentrations of H2S and HS- and other sulphur compounds

4. the partial pressure of oxygen

5. the pH solution 

The methods of current analysis used determined the total content in H2S and HS- and polysulphurs existing in mg H2S/ l.

The expression of the results in mE/ l anions of sulphur in state of global oxidation –2 is the most correct.

The waters with a high content of H2S and a pH at the source between 7,4 and 7,6 have a higher percentage of extractable colloidal sulphur, than the one corresponding to H2S and HS- and even S2O32-, indicating the presence of some anions richer in sulphur, such as polysulphurs.

In case of polysulphurs absence, the ratio between H2S and HS- can be estimated by calculation, depending on the temperature and pH.

The redox phenomena can be determined by the oscillations of the S2O32- anions, the increase of concentration in SO42-, and the deposits of colloidal sulphur.

The tendency of the sulphur compounds oxidation from the state of oxidation –2 (H2S, HS-, polysulphurs) is closely correlated with their concentration and partial pressure of the oxygen, an intensification of the phenomenon of oxidation can be observed in more alkaline pHs.

The capsulated probes, without the presence of an air volume above the liquid, have a lower tendency of oxidation and this is not more limited in colloidal sulphur, but also in superior products of oxidation (oxygenated anions of sulphur). 

# Meeting report: COMPLEX REHABILITATION TREATMENT IN OSTEOARTHRITIS

**Authors:** R Popovici, I Petrusca, L Cioc, S Neagoie

**Introduction.** The pathological changes in osteoarthritis are a consequence of a wide variety of etiological factors that ultimately lead to the alteration of the articular mechanical function and the structural damage of the articular cartilage. It occurs in more than 80% of the people over 55, becoming a public health problem. The most common localizations having a direct relationship with age are the following: the joints of the hands, the hip, the knee, and the interposed joints of the spine.

**Objectives.** Through the complex methods of treatment provided by rehabilitation, a trial is made to combat pain and inflammation, ensure good joint stability, preserve articular mobility within functional limits, correct the possible deformations and alterations of articulation, preserve and/ or restore walking, the desiderate of improving functionality. However, the most challenging part is the education of the patient and family.

**Material and Methods.** Over 50 treatment methods have been described, 20 of them being applied in all the identified practices. There are no agreed and universally applied guidelines. Moreover, there are no significant differences in pain control between non-pharmacological and pharmacological measures and there is a consensus regarding 25 therapeutic recommendations. 

**Results.** Arthrosis is defined as chronic pains of the articular joints due to joint wear, respectively destructive cartilage damage, plus proliferative changes of the adjacent bone. The optimal management of the hip and knee osteoarthritis is a combination of non-pharmacological treatment and pharmacological treatment.

# Meeting report: IMPROVING ARTICULATION AND MUSCULAR STRENGTH THROUGH EXERCISES USING ELASTIC RESISTANCE IN ELDERLY PEOPLE

**Authors:** A Oltean, N Calotă

Mobility is a fundamental issue that needs to be addressed to maintain the physical independence of people of the third age.

The purpose of this paper was to apply an exercise program using elastic resistance exercises of different tensions to improve joint mobility, muscle strength and other mechanisms disturbed by the aging process in elderly people.

The two subjects enrolled in the study were O.F., female, 60 years old (subject # 1) and O.O., male, 65 years old (subject # 2). This study was conducted at the patients’ home for 6 months (December 2016-May 2017), three times a week, during which each subject achieved the individualized elastic resistance exercise program and strictly followed the guidelines for exercising. The program aimed to improve joint mobility and muscle strength in the lumbar spine, knee joint and scapular-humeral joint in each of the two subjects, also taking into account associated pathologies.

Subject # 1 totaled 48 hours of training, and subject # 2, 36 hours of training.

The tests applied were the following:

The “Sit and Reach Test” test - to determine the spine mobility in the anterior plane. 

The articular balance was achieved by measuring the amplitudes of motion in the joints using the goniometry in all possible movement directions.

“Sorensen Biering” test - to identify muscle strength of body segments.

Following the interpretation of the results obtained in the two tests, it was observed that the subjects of the research improved the articular mobility of the dorso-lumbar spine, the scapulohumeral joint in the case of subject # 1, the knee joint in the case of subject # 2, the force of the extensor muscles of the lumbar spine, the flexing muscles and the extension of the knee, and the scapular-humeral joint muscles, which confirmed our hypothesis.

# Meeting report: THE ROLE OF THE RECOVERY PROGRAM IN THE TREATMENT OF MULTIPLE SCLEROSIS

**Authors:** IS Neagoie, D Cinteză

**Introduction.** Multiple sclerosis is a chronic central nervous system disorder characterized by focal inflammation and demyelination episodes with multiple localizations and a parallel axonal degenerative process, in a person with genetic susceptibility to the disease. It is the most common neurological disorder of the young adult, causing major disabilities.

**Objective.** Presentation of the benefits of the individualized recovery program in the treatment of patients with multiple sclerosis.

**Material and method.** According to the latest studies, treatment should be initiated before the irreversible loss of a function. From the perspective of improving the quality of life, symptomatic and recovery, treatment is just as important as modifying the treatment according to the disease progression. It is adapted according to the clinical stage of the disease and the degree of disability characteristic to each patient, having the aim to improve the quality of life and maintain social integration.

**Conclusions.** Exercise is part of the therapeutic management of multiple sclerosis. Effects are especially beneficial for disability and quality of life. Exercise should be dosed individually and performed with some caution. There has been no effect of favoring the disease progression through physical training.

# Meeting report: MULTISYSTEM ATROPHY A PARKINSON-LIKE SYNDROME

**Authors:** O Naglabeala, I Petrusca, D Cinteză

A case report regarding a patient, who was late diagnosed with a rare and potentially severe disease, ignored by patient himself and family, with a quick clinical and functional deterioration in last two months, with many complications and associated conditions, who needed an efficient rehabilitation program, but did not meet the criteria for rehabilitation.

# Meeting report: REHABILITATION OF A CASE WITH ALGOFUNCTIONAL SEQUELAE AFTER PELVIC GIRDLE FRACTURE BY USING NATURAL ENVIRONMENTAL FACTORS SPECIFIC TO TECHIRGHIOL RESORT

**Authors:** E Cristescu, LE Stanciu, M Minea

Pelvic girdle fractures are the consequences of modern times with excessive motorization and speed, with an incidence between 0.3% and 6% of the total fractures and 20% in the case of polytrauma. They are frequently accompanied by other bone or visceral lesions that associate important bleeding and high mortality, but also late complications: chronic pain, inferior limb inequalities, sexual dysfunction. When not surgical, the therapeutic decision involves prolonged immobilization with side effects such as muscle mass loss, joint stiffness, decreased exercise tolerance, which greatly complicates the physician’s recovery task.

We presented the case of a 24-year-old patient, who, in February 2017, suffered a polytrauma-related ski accident at the pelvic girdle level - left ischium and pubis ram fracture, comminuted fracture of the iliopubic ramus, fracture of the sacrum, 18 mm pubis disjunction, subperitoneal hematoma - treated conservatively by immobilization for 2 months before entering Techirghiol Balnear and Rehabilitation Sanatorium, for the recovery of the gait and motor deficit installed on the left lower limb.

Following a serious accident in the Azuga resort, the patient was carried away, without benefitting from specialized investigations, by two medical units in Prahova County before being transferred to S.C.J.U.C. where a non-surgical treatment approach was decided. The patient stayed at home for two months, during which, the hypotrophy of the lower left leg aggravated, being unable to perform the ADL, and worsening the quality of life. Following a neurological consultation by a visit at home, he was admitted for treatment in Techirghiol Balnear and Rehabilitation Sanatorium, where he benefited from balnear-physical kinetic complex treatment using the specific natural environmental factors: sapropelic mud and salt water from Techirghiol Lake, where he regained total walking independence and reintegration into his activity.

There are many indications of recovery treatment in Techirghiol Balnear and Rehabilitation Sanatorium, such as degenerative and inflammatory rheumatic diseases, lesions of central and peripheral nervous system, dermatological, gynecological, and peripheral vascular diseases, etc., most of them with a relief of more than 60% of the symptomatology. Of these, the best and fast results were observed in post-traumatic sequelae mainly due to the immersion in the very salty water of Techirghiol Lake that facilitates orthostatism and walking with the elimination of body weight and increasing muscle strength by opposing resistance to movement.

# Meeting report: PLANTAR FASCIA – PATHOLOGY AND REHABILITATION

**Authors:** AR Romila, L Draghescu, H Lazarescu

**Affiliations:** * Clinic II, National Institute of Physical Medicine & Rehabilitation, Bucharest, Romania

The ankle and foot represent together an anatomical and functional unit designated to assure stability and mobility simultaneously, being the terminal level of the locomotion. Painful and dysfunctional syndromes of the foot are frequently met in everyday practice and their etiology varies a lot. Among the multitude of pathologic entities of the foot the following are included: rheumatic diseases, degenerative problems, neurological disorders, common trauma (sprains, wrenches, fractures), and also pathology caused by sustained dynamic and static excess demand.

The most common reason for inferior heel pain, plantar fasciitis, is a degenerative process of the plantar fascia caused by excessive loading. It can be associated with sports (running, dancing), obesity and prolonged standing. It can also be caused by systemic inflammatory conditions. Some anatomic features seem to make plantar fasciitis more likely to occur (pes planus, pes cavus, heel pronation, incorrect footwear).

The rehabilitation protocol includes three phases. Because of the high complication rate and the self-limited nature of over 90% of the patients after 6 to 10 months of conservative therapy, of all phase treatments for 12-18 months, operative indications are extended to failure. 

Complete or partial rupture of the plantar fascia may occur in jumping or running sports, though it was not commonly reported in the literature. It is frequently misdiagnosed as an acute flare-up of plantar fasciitis. 

Athletes, who continue their activities in spite of the symptoms and neglect the treatment, will confront with an increase of pain most of the times, until it interferes with everyday activities, and also the irreversibility of the degenerative process.

# Meeting report: LACU SĂRAT RESORT

**Authors:** DA Dumitrascu, H Lazarescu

Lacu Sărat resort is located in the north-eastern part of Bărăgan plain, belonging to Chiscani county and being connected to Brăila municipality by an all-seasons tram line, which has been operative for more than a century. 

The lake, created by an older Danube flow, has a depth varying between 0.6 and 1.80 meters, and its entire bottom is covered by a highly mineralized therapeutical mud. It is considered that here there is one of the most valuable vegetable slimes in the country and waters with the highest saline concentration. 

A hundred years ago, this place was known both in the country and in Europe as one of Romania’s luxury resorts, being a preferred destination for weekends. As early as 1875, one could find here a true balneary and climatic complex, with an establishment for warm and cold baths, soft water showers, individual and common bath facilities, “hydrotherapy cabinets, physiotherapy, massages and electrical baths” and also “a midwife for gynecological treatment”. Currently, the balneary complex benefits from a treatment facility with a capacity of 250 people, having a pool for water therapy exercises, sauna, and gym. Additionally, amenities of free balnear services, with sun- and mud-baths are provided.

# Meeting report: KNEE SPRAIN MANAGEMENT

**Authors:** D Poenaru

Knee sprain diagnostic is based on clinical and imagistic evaluation. IRM was classically recommended for precise lesion identification, but, recently, sonography gained a place due to dynamic screening, repeatability, and cost-effectiveness. For medial collateral ligament, sonography identifies the lesion type, the periarticular structures integrity, being an important diagnostic step.

Medial sprains are treated in the rehab department, at least of grades 1 and 2, and, with greater frequency, of grade 3, being an alternative for surgical approach. Conservative methods are efficient, as rest, compression, physical therapy, and kinesiotherapy. 

The rehab program is patient oriented, depending on the state progression and sonographic evaluation.

# Meeting report: PREVENTION OF FALLS IN PATIENTS WITH PARKINSON’S DISEASE

**Authors:** L Draghescu, AR Romila

**Affiliations:** * Clinic II, National Institute of Physical Medicine & Rehabilitation, Bucharest, Romania

Parkinson disease is a progressive, neurodegenerative movement disorder, characterized by tremor, bradykinesia, rigidity, postural instability. Incidence of falls is 45-65% annually and they are caused either by intrinsic factors (postural changes, uncontrolled gait, vision changes, orthostatic hypotension, multiple medications) or by extrinsic factors (environment).

Falls are associated with serious complications including fractures (hip fracture), head trauma, and other injuries. Falls can trigger a vicious circle that aggravates the patient’s condition even more: fear of other falls and injuries reduces the subject’s mobilization that in turn causes osteoporosis, loss of independence, decreased fitness, and reduced participation in simple daily activities. 

The most important aspect is fall prevention and besides optimal medication, the physical therapy is the most effective aid for patients with Parkinson disease. Early intervention should be the focus, increasing ROM, improving coordination of movement, and improving posture and functional abilities. Balance training can prevent falls. Improving physical capacity with aerobic training, strength and flexibility exercises may reduce symptoms as well as improve the patient’s general well-being and quality of life.

# Meeting report: ASPECTS REGARDING CARDIO-VASCULAR PATHOLOGY IN PEOPLE WITH TRAUMATIC SPINAL CORD INJURY

**Authors:** L Neacsu, H Lazarescu, A Dima, A Cristea

**Affiliations:** * Clinic I, National Institute of Physical Medicine & Rehabilitation, Bucharest, Romania

**Introduction.** Data published in the last years showed that morbidity and mortality rates due to cardio-vascular pathology are higher than those due to renal or pulmonary dysfunctions, this being considered a very important cause of death in patients with spinal cord injuries.

The main major risk factors for developing this kind of pathology – overweight, dyslipidemia, metabolic syndrome, and diabetes have a high prevalence and it is necessary that an adequate treatment for these problems is the main part of the therapeutical management of these patients, in order to minimize the risk of developing severe cardio-vascular problems as much as possible. Due to spinal cord injuries, the autonomous nervous system dysfunctions, as well as the significantly low energetic daily consumption in this category of patients, also contributes to a higher risk of developing this kind of pathology.

**Purpose.** The update of the main factors that determine a high cardio-vascular risk in patients with spinal cord injuries, although many of them are young, as well as the therapeutical management that has to be considered in order to minimize the mortality rates due to these problems as much as possible.

**Methods.** International literature materials and data from our practical experience regarding the complex therapeutical approach that is necessary for this category of patients.

Treatment management includes the education of the person, medication, physical exercise with the indications and limits imposed by the basic pathology.

**Result.** Long-term survival and evolution of people with spinal cord injuries depends on the onset of cardio-vascular problems and it is necessary to prevent and treat them as early as possible in order to decrease the rate of mortality due to these reasons.

**Conclusions.** Treating risk factors and promoting physical activity in different ways, taking into account that the clinical particularities are mandatory, in order to decrease the chances of developing multiple severe complications and also to improve the quality of life in this category of patients.

# Meeting report: SPORT AND EPILEPSY

**Authors:** S Diaconescu, D Cinteza

**Affiliations:** * Neurologic Rehabilitation Department, National Institute of Physical Medicine & Rehabilitation, Bucharest, Romania

Sport only has beneficial effects for epileptic patients and their mental and social health. It probably even has the effect of reducing the likelihood of seizures by tackling the anxiety and depression that are often associated with-and exacerbate-the condition. It also has an impact on patients’ waistlines, as some AEDs increase their appetite, as well as helping to tackle osteoporosis that is associated with AEDs. It is exceptionally rare for seizures to be triggered by sport.

Epilepsy is still sometimes a source of fear because of the aura that surrounds it, the fact that there is no sign of the condition prior to a seizure, and the unpredictability of seizures.

There is almost no reason for patients not to play football, quite the opposite. However, others should be made aware of the situation, in the interests of honesty and to avoid any panic.

Denying an epileptic the opportunity to play increases both exclusion and stress.

# Meeting report: GOUTY ARTHROPATHY IN THE KNEE WITH SECONDARY KNEE OSTEOARTHRITIS

**Authors:** D Dimulescu, G Chiriţi, RG Scarlet, L Drăghescu, A Romila, V Tuțu-Pața

**Affiliations:** * “Carol Davila” University of Medicine and Pharmacy, Bucharest, Romania; ** National Institute for Physical Medicine, Recovery and Balneoclimatology, Bucharest, Romania

**Objectives.** The purpose of this case report was to measure the role of a complex program, which included physical-kinetic treatment for a patient who suffered from acute gout arthritis with secondary knee osteoarthritis. 

**Materials and methods.** We presented the case of a 62-year-old woman, who was hospitalized in our clinic for combined symptoms including pain, mainly inflammatory, the edema of small hand joints (mainly at the right hand) and at the ankle, morning stiffness (for more than an hour), associated knee pain, moderate functional impairment–difficulties at walking on slope without additional help.

From her personal medical history, we mentioned the following: in 2015, the patient had an acute gout arthritis with secondary knee osteoarthritis for which she received NSAID, urate-lowering therapy and antialgics. At the clinical exam we observed the following: pain and at palpation, at weight load and movements of knees, at patellar mobilization bilaterally, bilateral liparthrosis, active flexion at 70 degrees, passive flexion at 80 degrees, irreducible flexum at 10 degrees bilaterally; spontaneous TT joint pain bilaterally accentuated by weight load and movements, edema of the proximal and distal interphalangeal joints, bilaterally, metatarsophalangeal and of the interphalangeal joints of the I-V fingers bilaterally, gout tophi at the left elbow, of the distal interphalangeal joints of the II and III fingers of the right hand, metatarsophalangeal joints and interphalangeal of the halluces bilaterally. The complex rehabilitation program used: medical treatment, antialgic electrotherapy with myorelaxation effect, cryotherapy on the small joints of the hands (mainly on the right hand) and metatarsophalangeal joint and interphalangeal of the I-V fingers, sedative massage for the cervico-dorso-lumbar vertebral column; kinesiotherapy with postural therapy exercises for the lower limbs.

**Results.** After the application of the treatment, the following results were the following: VAS reduced from 10 points at the admission to 6 points at the discharge; the improvement of the inflammatory syndrome of the small joints of the hands with 75%, improvement of the inflammatory syndrome at the metatarsophalangeal joint, the interphalangeal joints of the I-V fingers bilaterally with 80%, improvement of the active flexion of the knee at 80 degrees and of the passive flexion at 90 degrees and the improvement of the walking pattern.

**Conclusions.** The rehabilitation program benefited from the algo-dysfunctional and inflammatory syndrome of the small joints of the hands and of the metatarsophalangeal and interphalangeal joints of the I-V fingers bilaterally, improvements of the active and passive flexum of the knees being registered.

**Keywords:** knee osteoarthritis, gout, arthritis

# Meeting report: SECONDARY KNEE OSTEOARTHRITIS WITH RIGHT KNEE ANKYLOSIS. MULTIPLE POSTRAUMATIC SEQUELAE

**Authors:** G Chiriţi, D Dimulescu, RG Scarlet

**Affiliations:** * “Carol Davila” University of Medicine and Pharmacy, Bucharest, Romania; ** National Institute of Rehabilitation, Physical Medicine, and Balneoclimatology, Bucharest, Romania

**Objectives.** The purpose of this study was to evaluate the role of a complex programme, which includes a physical-kinetic rehabilitation treatment for a patient with secondary posttraumatic knee OA, with right knee ankylosis.

**Materials and methods.** We presented the case of a 68-year-old patient, who was hospitalized in our clinic for mixed pain, mainly mechanical pain at the hip and knee joints, (mainly on the right), mechanical pain of the right forearm, marked functional impotence – difficulties in walking, with right knee ankylosis, with the use of a walking stick. The following information were underlined from her personal medical history: the patient suffered a car accident with multiple trauma (2014) such as ischiopubic bilateral fracture, left sacrum bone, multiple fracture of the intra-supracondylar right femur, fractures of the tibial spine and of the left tibial plateau, the fracture of the right patella, of the right humeral neck surgically treated, distal fracture of the right radius, treated by orthopedic surgery (metallic OS), with the removal of the osteosynthesis material after 2 years. In 2016, the patient suffered a corticotomy of re-fixing of the right knee, followed by the knee ankylosis.

The following could be seen at the local exam: increase of thoracic kyphosis, reduction of the lumbar lordosis, paravertebral muscular CDL contracture, mainly on the right side, Schober = +3 cm, IDS – patellar; pertrochanteric bilateral pain worsened by mobilization and weight load, 70 degrees flexum (right), 75 degrees (left), 20 degrees abduction (bilateral); spontaneous pain at the palpation an weight load of the knees bilaterally; sounds at the patellar mobilization; right knee ankylosis; left knee: active flexum at 60 degrees, passive flexum at 70 degrees, irreducible flexum at 10 degrees; left foot longer than the right foot with 1 cm; difficulties at walking, with auxiliary help.

The complex rehabilitation program used the following: pharmacological treatment; electrotherapy with antialgic and myorelaxation effect – Diapulse on the right knee and right forearm, sedative massage of the vertebral cervico-dorso-lumbar vertebral column; kinesiotherapy with postural therapy exercises for the improvement of the mobility on the inferior limbs; exercises for walking and coordination.

**Results.** The following were observed after the treatment: the VAS scale has lowered from 8 points at the admission to 6 points at the discharge; the improvement of the algo-dysfunctional syndrome of the hip and of the left knee: the improvement of the active flexum to 65 degrees and of the passive one to 75 degrees, the improvement of the walking pattern.

**Conclusions.** The rehabilitation program has improved the algo-dysfunctional syndrome at the level of the inferior left limb.

**Keywords:** knee ankylosis, secondary knee arthritis

# Meeting report: STUDY ON THE EFFECTS OF THE MF ON A PATIENT GROUP WITH MUSCULAR CONTRACTURE WHICH APPEARED DURING THE CHRONIC LUMBAR PAIN

**Authors:** EM Dragosloveanu

**Affiliations:** * “Carol Davila” University of Medicine and Pharmacy, Bucharest, Romania; Medical Rehabilitation Clinic – Orthopedy and Traumatology; National Institute of Rehabilitation, Physical Medicine and Balneoclimatology, Bucharest, Romania

The study was performed on a group of 48 patients (aged 20-69 years), with chronic lumbar pain and paravertebral lumbar contracture, who had a treatment with MF applied in the lumbar area, daily, for 10 days, with constant medium frequencies and a variable frequency between 0 and 100 Hz. The study was prospective.

The therapeutical associations that could produce muscular relaxation were excluded. The association of the anti-inflammatory, analgesic or decontracturant drugs, and other rehabilitation procedures (electrotherapy, massage, kinesiotherapy, and bed rest) was not permitted.

No other procedure was performed to the lumbar area. 

The contracture and the lumbar pain were appreciated based on some subjective and objective criteria. The results obtained while applying the MF were good and very good in 42 patients, in whom the muscular contracture and the pain disappeared after 10 days of treatment.

Most of the patients appreciated the decontracturant and myorelaxation effect of the procedure. The application method with constant “medium” frequencies (50 Hz) has proven to have decontracturant effects and the variable frequencies rhythmically between 0-100 Hz, produce an active deep micro massage of the striate muscle, regulate the modified pathological tonus of the tissues, with benefic effects in muscular contractures, and also in the pain caused by these. 

This study, although performed on a small group of patients, showed the efficacy and the well tolerance of the medium frequency in the treatment of chronic lumbalgia.

# Meeting report: THE EVALUATION AND THE WALKING TRAINING OF PATIENTS WITH SNMC

**Authors:** V Suciu, L Cioc, D Cinteza, L Rarinca, T Olariu, I Burlacu, H Lazarescu

**Affiliations:** * National Institute of Physical Medicine & Rehabilitation, Bucharest, Romania

The training on conveyer is often used in the rehabilitation of walking in patients with neurological diseases starting from the supposition of the neuroplasticity stimulation by influencing the activity of the spinal centrum that generates movement schemes (spinal CPG) and by the use of the principle of repetition in the learning and consolidation. Despite these, this type of training is not fully standardized, indications are not clear, and some scientists question the oriented specificity of this type of training.

The purpose of this study was to decide the indications and the efficacy of the training with the use of the robotic tool in the walking rehabilitation in patients with neurological diseases.

This robotic tool contains 4 modules: “Gait Analysis” – the module for walking analysis and for the creation of walking patterns for the patients; “Eye Gait” - the active training for the superior limbs in the treatment; “Virtual Reality” – the module that places the patient in a familial environment; “Games” – the “games” interface, which allows the stimulation of the cognitive functions and the rehabilitation of the equilibrium functions of the patients in a complex and unified way. 

**Materials and methods.** The research, which is still in progress, is of prospective type. The patients included were with neurological diseases, capable of walking at least 5 steps with or without support, no matter the spasticity degree. Patients were evaluated regarding the demographical, general medical history, neurological and functional aspects by registering with the robotic tool the walking parameters at the beginning of the study and after 10 training lessons.

**The results** allowed the prediction of the indications for this type of treatment, of the criteria the patient must have so that the treatment is efficient. The study also presented the standard applied methodology, with individual variations regarding the suspension degree and the speed.

**Conclusions.** The walking rehabilitation method using the robotic tool in patients with neurological diseases can be beneficial if the application indication is adapted to the specific needs of the patients. The mechanism it uses is that of stimulating the neuroplasticity and the result can be an unwanted one, if the neuroplasticity is not oriented towards the final purpose, which is adapted to each individual.

# Meeting report: VITAMIN D - A NEW SCIENTIFIC APPROACH

**Authors:** E Craiu

Vitamin D is actually a hormone produced by the skin. Observational studies have found associations between low levels of vitamin D in the blood and an increased risk for cardiovascular diseases.

Vitamin D is a group of soluble vitamins in scientific research, with two main physiological forms: vitamin D2 (ergocalciferol) and vitamin D3 (cholecalciferol). Although vitamin D is directly involved in bone metabolism, the target organs on which its active derivatives act are the following: intestine, bone, kidney, parathyroid, and muscle.

The classical research has demonstrated several objective features of vitamin D deficiency and numerous recent studies have used plasma 25-OH-D as a measure of vitamin D status.

More recently, clinical research has led to the observation that about 20% of the side effects of statin treatment are related to fatigue and muscle pain, but also to low vitamin D blood levels. 

The subject is new and opens a vast area of clinical research and specialist studies.

Larger robust clinical trials will be needed to understand the complex ways in which statins and vitamin D might affect each other’s properties and actions.

Vitamin D supplementation might be a safe and affordable treatment option in polycystic ovary syndrome (PCOS) or might support couples who want to have children.

In a study by Shantha and colleagues, patients with vitamin D levels in the lowest quartile had a 1.21 times increased risk for statin-induced myalgia compared to those in the highest quartile. Levels of 15 ng/ mL or lower positively predicted the development of myalgia in statin users.

In summary, these two studies provided interesting observations regarding vitamin D, statins and atherosclerosis/ CIMT. The mechanisms for a vitamin D amplification of statin effect requires further study to test this hypothesis. The participants with higher vitamin D levels did not have lower cholesterol, decreased disease activity, or lower hsCRP levels. Vitamin D may modify a variety of biological pathways that could affect atherosclerosis and the effectiveness of statins. Although this observation has a potential clinical relevance, the wide spread screening for vitamin D deficiency and the increasing use of vitamin D supplementation may make it difficult to study the effects of vitamin deficiency in the future.

This study provided insight to the potential relationship between vitamin D concentrations and statin-induced myopathy. The findings were that patients with documented statin-induced myopathy had significantly lower vitamin D concentrations and the majority of these myopathies were observed in patients with a vitamin D <32 ng/ mL.

In a clinical setting, vitamin D status may be considered a modifiable risk factor for muscle-related adverse effects of statins, and supplementation of vitamin D (particularly when ≤ 20 ng/ mL) may improve statin tolerance. Well-designed, prospective research could be warranted to evaluate this hypothesis.

Statin intolerance because of myalgia, myositis, myopathy, or myonecrosis associated with low serum vitamin D can be safely resolved by vitamin D supplementation (50,000-100,000 units/ week) in most cases (88-95%).

A rare side effect known as myositis is defined as muscle symptoms in association with a substantially elevated serum creatine kinase (CK) concentration. Creatine kinase is the enzyme released from damaged muscle cells, and CK elevations > 10× the upper limit of normal (ULN) occur in 1 per 1000 to 1 per 10 000 people per year, depending on statin, its dose and the presence of other risk factors.

Larger robust clinical trials will be needed to understand the complex ways in which statins and vitamin D might affect each other’s properties and actions.

It is presently premature to recommend vitamin D supplementation as treatment for statin associated muscle complaints in the absence of low vitamin D levels, although such supplementation could be tried in patients with deficient or reduced vitamin D levels.

# Meeting report: NEW FAST METHOD OF DETERMINING THE QUALITY OF MINERAL WATER AND THERAPEUTIC MUD IN SOME BALNEOLOGICAL RESORTS IN ROMANIA

**Authors:** AP Khachatryan

**Affiliations:** * Prof. Khachatryan’s Alternative Medicine Clinic, Novosibirsk (Russia), Astana (Kazakhstan)

The healing properties of any mineral water and mud are determined primarily by their mineral composition, i.e. those organic and inorganic substances that are dissolved in them. However, their general physiochemical indexes are no less important. To this end, to date, only pH is used - a unit for measuring the activity of hydrogen ions in water, quantitatively expressing its acidity.

However, in our opinion, there are more important indicators that characterize the physiochemical properties of mineral waters and therapeutic muds, which are not taken into account when determining their quality. This is the *oxidation-reduction* or *redox potential* and the concentration of active hydrogen.

The main processes that ensure the vital activity of any living organism are the oxidation-reduction reactions, i.e., reactions that deal with the transfer or attachment of electrons. Substances that include atoms that attach electrons during the reaction are called *oxidizers*, and substances that transfer electrons are the *reducing agents*. The energy released during these, engages in maintaining homeostasis (vital activity of the body) and regenerating the body cells. One of the most significant factors in regulating the parameters of oxidation-reduction reactions occurring in any liquid medium is the activity of electrons or, in other words, the ORP of this environment (ORP). The redox potential is determined by the electrochemical method and is expressed in *millivolts*. The ORP values can be either positive or negative. Positive values are characteristic for a medium with oxidizing properties, and negative values for an environment with reducing properties.

Normally, the ORP of human blood (measured on a platinum electrode referred to a silver chloride electrode) ranges from -70 to -100 millivolts (mV), that is, the internal environments of the human body are in a restored state. According to World Health Organization, the drinking water should not exceed the ORP of + 40 mV. ORP of ordinary drinking water (tap water, bottled water, etc.), measured in the above manner, is usually greater than zero, and usually ranges from +200 to +300 mV. These differences in ORP of the internal environment of the human body and drinking water mean that the activity of electrons in the internal environment of the human body is much higher than the activity of electrons in drinking water.

When drinking water penetrates the human tissues (or other living creature) body, it takes the electrons away from cells and tissues. As a result, the biological structures of the body (cell membranes, cell organelles, nucleic acids and others) are subjected to oxidative destruction.

If the drinking water consumed by the body has an ORP close to the ORP value of the internal environment of the human body, then the electrical energy of the cell membranes (the body’s vital energy) is not consumed to correct the activity of water electrons and water is immediately absorbed because it has biological compatibility in this parameter.

If drinking water has a more negative ORP than the ORP of the internal environment of the body, it feeds it with this energy, which is used by cells as an energy reserve of the body’s antioxidant defense against adverse environmental influences.

Another significant and almost unexplored parameter is the index of *active hydrogen* or *hydride-ion*. What is it? H- is a physical system consisting of one proton and two electrons. In other words, it is a form of hydrogen, in which the atom has an extra electron, which gives it a negative charge. It acts as an antioxidant in various biological systems and is found in virtually all fresh, raw products of vegetal and animal origin. Moreover, it is the exact parameter that determines the negative redox potential of the fluids of our body.

There are not many natural water sources on planet Earth that have a negative redox potential and a corresponding concentration of active hydrogen. Among people living in such areas, a greater number of centenarians was noted.

Romania is a country famous for its balneology resorts. The country concentrates almost a third of all mineral wells discovered in Europe. Their healing properties have been known for centuries. It should be noted that in one location there might be several types of salt-rich natural water and therapeutic mud. Considering that the above parameters were not taken into account when determining the quality of mineral waters and therapeutic mud, it was curious to research the redox potential and the concentration of active hydrogen in some mineral springs and therapeutic muds from the rich, natural sources of the country.

Studies were carried out at four balneology resorts in Romania: Borsec, Covasna, Băile Tușnad, Techirghiol. The redox potential was measured on a portable instrument ORP-meter HI 98201 by HANNA (Germany); Active hydrogen - on a portable device TRUSTLEX ENH-1000 (Japan). The results of the studies are reflected in **Table 1**. The measurements were calculated directly from sources (wells) as per table below:

**Table 1:** Oxidation-reduction potential (ORP) and active hydrogen indicators (H-) of mineral water in some balneology resorts in Romania

CITY NAME	WELLS NAME	WELLS №	ORP (mV)	H- (ppb)
BORSEC	Untitled	3	-135	122
	Untitled	5	-150	170
	Untitled	6	-380	340
	Untitled	10	-175	130
	Untitled	11	+40	0
COVASNA	Elvira	Left	-254	330
	Elvira	Right	-238	280
	Untitled	Under the bridge	-194	214
	Untitled	Montana st.	-73	16
	Untitled	By the school	-162	138
BĂILE TUȘNAD	Untitled	1	+135	0
	Untitled	3	+130	0
	Untitled	4	+111	0

Studies of the mineral water at Borsec resort showed the following: at sources No. 3, 5, 6, 10, the water had a negative ORP and a high percentage of active hydrogen. Moreover, the water at source 6 was almost 2 times higher in terms of its parameters, the indexes in sources 3, 5 and 10. Water in source 11, had a positive ORP, and active hydrogen was absent in ppb values. Similar good results for the indicators of ORP and active hydrogen were obtained at the sources of Covasna resort. However, at Băile Tușnad resort, the ORP in all three studied sources had positive values, and active hydrogen was absent in all of them.

Investigating the mineral composition of all the sources described above, we did not find any significant differences between the elements, the presence or absence of which could affect the hydrogen index and the ORP values.

A certain correlation was revealed between them when comparing the indicators of ORP and active hydrogen. The lower the ORP value, the higher the pH and vice versa.

The indicators of ORP and active hydrogen of local therapeutic mud and water from the lake were studied at Techirghiol resort. Studies have shown that the ORP of the water from the lake was +120 mV, H- = 0 ppb. ORP of mud = -693 mV, H- = 944 ppb. When mixing mud with water from the lake in certain percentages, the following results were obtained: 2% of the mud: ORP = -526 mV, H- = 546 ppb; 10% of mud: ORP = -463 mV, H- =338 ppb.

Investigating the ORP of therapeutic mud in different countries, it was found that they all had a negative ORP. This was due to the vital activity of certain microorganisms living in it. Moreover, the lower the ORP of mud, the more evident were its healing properties. 

**Conclusions.** 1. Indicators of ORP and active hydrogen can serve as a new fast method for determining the quality of mineral waters and therapeutic mud;

2. A certain correlation was found between the ORP indicators and active hydrogen: the lower the ORP, the higher the hydrogen index.

3. Mineral waters with low ORP and high hydrogen index possess restorative, antioxidant properties and, respectively, have a greater therapeutic effect on the body.

4. The therapeutic effect of mud is determined by the values of its ORP and the index of hydrogen. The lower the ORP and the higher the hydrogen index, the better their therapeutic properties.

# Meeting report: THE MANY FACES OF SPA – SPONDILARTRITIS BEYOND ARTICULAR MANIFESTATIONS. THE IMPACT OF TREATMENT ON THE PATIENTS’ QUALITY OF LIFE

**Authors:** D Oprea, M Minea

**Affiliations:** * Techirghiol Balnear and Recovery Sanatorium, Techirghiol, Constanța, Romania; ** Ovidius University, Constanța, Romania

**Introduction.** Spondyloarthritis (SpA) is defined as an inflammatory arthropathy characterized by the involved sacroiliac joints and the association with HLA-B27 alleles. Many names such as the following were proposed for this group of disorder: seronegative spondyloarthropathies, chronic inflammatory seronegative rheumatic diseases, or seronegative inflammatory arthritis.

In 1974, Moll and Wright introduced the term seronegative spondyloarthropathies, which refers to a group of seronegative inflammatory rheumatic diseases.

**Materials and methods.** The most important features are represented by the axial structures’ inflammation (especially sacroiliac joints), asymmetric oligoarthritis (especially at the lower limb, dactylitis (sausage fingers), enthesitis (the inflammation in the tendon, ligaments and fasciae insertion area). 

The other characteristic features of this heterogeneous group of diseases are the following: cutaneous and genital lesions, bowel and ocular inflammation, bowel and genital infections in recent medical history, familial cases of spondyloarthritis, increased values of the acute phase inflammation markers and the association with HLA-B27 antigen. Extra-articular manifestations can be associated at pulmonary, renal, cardio-vascular levels, which, depending on the organ’s lesion severity, can dominate the clinical picture.

**Conclusions.** With the purpose of starting an early and adequate treatment, the International Spondyloarthritis Study Group of ASAS established a sub-classification of the SpA disorders depending on the criteria of involved joint type in mainly axial SpA and mainly peripheral SpA, validating on this occasion the enrolment criteria in the two categories. 

The treatment of the patients with SpA with extrajoint manifestations is a challenge because it must prevent the disease progression with the development of ankylosis or of organ lesions, to alleviate pain and to increase the quality of life, reducing the morbidity and the mortality.

# Meeting report: RHEUMATOID ARTHRITIS PATIENTS MANAGEMENT. THE IMPORTANCE OF EARLY DIAGNOSIS AND T2T APROACH

**Authors:** M Minea, D Oprea

**Affiliations:** * Techirghiol Balnear and Recovery Sanatorium, Techirghiol, Constanța, Romania

Rheumatoid arthritis (RA) is a chronic inflammatory systemic disease with unknown etiology and autoimmune pathology, characterized by articular damage with progressive, deforming, destructive evolution. 

The assessment of patients with RA must include the following: clinical examination, the level of illness activity, the acute phase reactants (RF, ACPA), the early identification of bone erosions (using conventional radiography completed with USMS and MRI), the cardiovascular risk and other associated medical complications (neoplasms, fractures of fragility, gastrointestinal haemorrage).

The RA treatment has changed a lot during the last 15 years because the unraveling of the pathology mechanisms has lead to the introduction of therapy and the biologic agents. When the severe consequences of the illness and the necessity of preventing the articular destruction were found, they have modified the rheumatologists’ purposes. The management is more aggressive and involves the initiation of (DMARD’s) medication from the onset of the disease, the early use of biological therapy, and the “straight to the target” treatment strategy, that has as the ultimate goal and as quickly as possible the remission or at least the attainment of a low level of disease activity. There is no standardized method for the evaluation of the disease activity, since the composite indexes that are currently used (DAS 28, DAS 28 CRP, CDAI, and SDAI) contain a low degree of objectivity for the patient.

The concept of early PR has been more clearly defined during the last years as arthritis, with a period of evolution between 3 months and 1 year, during which the established medication had the greatest impact. It was formulated based on the 2010 ACR criteria, one of which is the number of affected joints whose identification is superior to clinical assessment.

As soon as the diagnosis is established, DMARDs should be initiated, Methotrexate being the first option. In case of contraindications or side effects, one of the other medicines in this class (Leflunomide, Sulfasalazine) might be chosen.

Hydroxychloroquine has a modest effect alone, but may be associated with another DMARDs to provide additional benefits to patients with cardiovascular risk. Glucocorticoids may have a “disease modifier” effect in early RA, being used as a link therapy associated with a DMARD’s to lower the disease activity, and then retrieve them by administering lower doses. High doses (30-60mg/ day) are prescribed in intensively active forms of disease and systemic manifestations, and intra-articular administration has a local efficacy and is not accompanied by general adverse reactions.

When MTX maximal treatment is not sufficient and the patient does not associate poor prognostic factors (high levels of FR and anti-CCP antibodies, early erosion and high disease activity), we may opt for the replacement with another DMARDs. If these factors exist, biological therapy is started by taking into account clinical experience, TNFα inhibitors being the first choice. The following molecules are currently used: Adalimumab, Infliximab, Etanercept, Golimumab, that most often perform an effective disease control. Other biological agents approved in PR therapy are Tocilizumab (anti IL6), Abatacept (T lymphocyte co-stimulant blocker), Secukinumab (anti IL17) and Rituximab (anti CD20). Although in clinical practice the fulfillment of the remission criteria is difficult, when it persists for more than 12 months, the decrease or the spacing of the therapeutic doses may be taken into consideration.

As it is a chronic disabling condition, it is important to inform the patient and his/ her involvement, together with the family, in making the therapeutic decisions to achieve the optimal management of this disease.

# Meeting report: TREATMENT WITH SAPROPELIC MUD IN PATIENTS WITH THYROID COMORBIDITIES - LIMITS AND BENEFITS

**Authors:** C Badiu

**Affiliations:** * “Carol Davila” University of Medicine and Pharmacy, Bucharest, Romania; National Institute of Endocrinology, Bucharest, Romania

Thyroid disorders are one of the most common endocrine diseases in Romania due to geoclimatic iodine deficiency, thyroid nodules frequency and specific autoimmune diseases. Often, they are considered as limiting factors for the application of balnear therapies, with adverse consequences for the therapy of underlying, invalidating diseases, requiring rehabilitation treatments.

In considering sapropelic mud therapy of osteoarticular diseases, several types of thyroid disorders, as well as comorbidities, should be considered. These are rarely really limiting factors. Thyroid nodules and implicitly papillary thyroid cancer have had an explosive epidemiology over the last decade, which has generated many management controversies followed by guidelines with a great dynamics. Autoimmune thyroid diseases have a broad functional spectrum ranging from subclinical pathology to clinically obvious hypothyroidism through Hashimoto’s thyroiditis and hyperthyroidism by Graves disease. Additionally, the endemic goiter, which is frequent in sub-Carpathian regions, Moldova and Ardeal, often adds to the frequency and necessity of a therapeutic attitude.

Of all the above, severe hypothyroidism (myxedema) and untreated hyperthyroidism (thyrotoxicosis) are contraindications to peloid therapy. The contraindications derive mainly from the combination of cardiovascular pathology and the effect of iodine on preexisting thyroid disorders. The absence of iodine in the Techirghiol sapropelic sludge composition is an important protective factor for thyroid pathology. Detailed randomized trials are required to delineate the limits and benefits induced by sapropelic mud therapy.

# Meeting report: IMPROVING FUNCTIONAL STATUS IN MULTIPLE SCLEROSIS PATIENTS BY PHYSICAL KINETIC, BALNEARY AND POSITIVE PSYCHOLOGY

**Authors:** A Bichis, L Cioc, H Lazarescu, D Cinteza

**Affiliations:** * National Institute of Physical Medicine & Rehabilitation, Bucharest, Romania

Multiple sclerosis is a progressive autoimmune inflammatory disorder that causes accumulation of neurological deficits over time.

The first part of the paper presented generalities about MS and drug treatment.

Physical-kinetic treatment presented together with the objectives and methodology of rehabilitation in the MS, revealed the realization of a short-term and long-term therapeutic plan, and a working model (adapted to the physiatric treatment).

The second part of the paper presented elements of spa treatment, spa resorts where MS patients can benefit from specialized treatment (aero-heliotherapy, HKTM, inhalotherapy, electrotherapy, etc.).

Also, the necessity of implementing the elements of positive psychology in the medical rehabilitation program was discussed to improve the functional status of patients with SM.

# Meeting report: REHABILITATION PROGRAMME FOR A PATIENT WITH VMT COMPLICATED WITH SYRINGOMIELYA

**Authors:** M Ganea

**Introduction.** Syringomyelia is the dilatation of the central spinal canal or the development of new cavities in the centro-medullary substance and it can also appear in vertebral trauma, as a result of the associated myelopathy. The mechanisms are incompletely known and the only method of treatment is the surgery for the drainage of the liquid cavities formed.

**Objectives.** Case report of a patient with vertebral cervical trauma complicated with syringomyelia above the lesion.

**Materials and methods.** A 49-year-old patient, suffered a vertebral cervical trauma and comminute fracture of C4 vertebral body in 2012, surgically treated and stabilized by corpectomy, titanium rachisynthesis and osseous graft, with motor tetraplegic secondary impairment at C5 neurological level, complete Frankel A lesion. In 2015, he started having pains and developed a decrease of the muscular force of the upper limbs. He was diagnosed with cervical C1-C4 syringomyelia. Surgery was performed in the same year. The patient followed a rehabilitation programme targeting the regaining of the muscular force at the upper limb and the improvement of the functional level.

**Results.** After repeated rehabilitation courses of treatment, the patient remained neurologically stable; the pain diminished and the patients’ quality of life increased.

**Conclusions.** The patient with vertebral trauma is a patient with multiple complications, possibly life threatening, who needs a complex therapeutic approach. Syringomyelia is one of these complications, which leads to the worsening of the myelopathy and of the pre-existing neurological deficits.

# Meeting report: BENEFITS OF SULFUR WATER TREATMENT IN THE EXTERNAL CURE

**Authors:** I Moise, A Dumitraşcu, G Sava, I Boroş

Mangalia spa resort is situated on the Black Sea coast.

The natural factors for the recovery of patients in Mangalia Balnear and Recovery Sanatorium were the following:

• Sulfur water that has special curative qualities, along with 

• Sea water, Techirghiol’s sapropelic sludge, and the usual physio-kinesiotherapy.

This natural resource is captured from two springs: F3 IBF and F4 IBF, being a sulphurous, chlorinated, bicarbonate, sodium, hypothermal water.

The physico-chemical and microbiological analysis of Mangalia sulphurous water has the following indications for external cure:

• Prophylactic - pre-arthritic conditions

• Chronic degenerative rheumatic diseases

• Chronic apathetic rheumatic conditions

• Biologically stabilized inflammatory rheumatic diseases

• Post-traumatic disorders of the locomotor system

• Chronic neurological disorders

• Respiratory disorders and ENT

• Chronic gynecological disorders

• Dermatological disorders

The external cure is used in the form of:

• Individual baths with sulphurous waters

• hydrokineotherapy in a pool of sulfuric water

• vaginal irrigation

• inhalation

# Meeting report: NINE MONTHS SURVEILLANCE AFTER SHOULDER DISPLACEMENT, ROTATOR CUFF TEAR, AND GLENOID FRACTURE – CASE PRESENTATION

**Authors:** N Campeanu, D Sandru-Campeanu, P Valenti, J Scanff, M Fermand, E Tarta-Arsene

**Affiliations:** * Department of Radiology and Medical Imaging - Group Hospitalier Est Francilien (Marne-La-Vallée), France; ** Inst. Parisien de L’Epaule - Clinique Biset – Paris, France; *** Kinesiotherapist; **** Centre d’Imagerie Galilee-Marne la Valee, France; ***** Department of Medical Imaging, “Victor Babes” Hospital, Bucharest, Romania

**Purpose.** The assessment and evaluation of morphological and functional impairment of shoulder structures and movements after a ski pivotal trauma, which led to rotator cuff tear and glenoid fracture.

Surgical procedure planning, outcome afterwards, and recovery plan to reduce the limitation of movements at this level after clinical procedures.

**Method.** The patient was evaluated clinically for patient satisfaction and pain relief scores. The fixation of the glenoid and humeral components, subsidence and notching were evaluated on radiographs. The indications were cuff tear arthropathy and fracture sequelae. Recovery plan to readjust the physiological functions of the shoulder was put into practice.

**Results.** After evaluating the Bankart lesion, surgical assessment and procedures were performed. We found an overall improvement in active forward flexion and abduction. No humeral loosening or subsidence was observed. Intra-operative fractures were reduced surgically and stabilized. 

The next step was to plan the optimal kinesio-therapeutic procedure in order to improve somatic performance of the shoulder. 

**Conclusions.** The clinical and imaging evaluation of a shoulder trauma and the proper planning of surgical procedures as well as the recovery procedures will definitely have excellent clinical results with no signs of loosening or subsidence.

# Meeting report: REVERSE SHOULDER ARTHROPLASTY WITH A SHORT METAPHYSEAL HUMERAL STEM

**Authors:** E Atoun, A Van Tongel, N Hous, A Narvani, J Relwani, R Abraham, O Levy

**Purpose.** Reverse shoulder prostheses have been gaining popularity in recent years. A short metaphyseal stem design will allow bone stock preservation and minimize stem related complications. We examined the clinical and radiographic short-term outcome of a short metaphyseal stem reverse shoulder arthroplasty.

**Methods.** Thirty-one patients, with a mean follow-up of 36 months (24–52) were clinically evaluated with the Constant-Murley score, patient satisfaction, and pain relief scores. The fixation of the glenoid and humeral components, subsidence and notching were evaluated on radiographs. The indications were cuff tear arthropathy (22), fracture sequelae (five), and rheumatoid arthritis (four).

**Results.** The average Constant score improved from 12.7 (range two to 31) pre-operatively to 56.2 (range 17–86) postoperatively. It rose from 13.5 to 58.3 in patients with cuff arthropathy, from 15.8 to 62.0 in revision arthroplasty, from 10.2 to 47.4 in those with fracture sequelae, and from 11.5 to 55.3 in patients with rheumatoid arthritis. The overall mean patient satisfaction score improved from 2.4/ 10 to 8.5/ 10 and the mean pain score improved from 0.8/ 15 to 12.5/ 15. We found an overall improvement in active forward flexion from 46.8 to 128.5° and from 41.6 to 116.5° in abduction. No humeral loosening or subsidence was observed. Two cases of grade 1–2 glenoid notching were reported. Overall, three intra-operative fractures did not affect the operation and healed without affecting the good results. There were five late traumatic periprosthetic fractures, only one of them requiring a revision surgery to a stemmed implant, the rest healing without surgery. Two early dislocations had to be revised.

**Conclusions.** The clinical and radiographic evaluation of a bone preserving metaphyseal humeral component in reverse shoulder arthroplasty was promising, with good clinical results, no signs of loosening or subsidence.

# Meeting report: ROLE OF THE PSYCHOLOGICAL INTERVENTION IN THE RECOVERY OF A PATIENT WITH ALGO-FUNCTIONAL SEQUELAE AFTER A POLIFRACTURE AT THE LEVEL OF THE ILIAC AND ISCHIUM BONES. CASE PRESENTATION

**Authors:** AM Dogaeas, E Cristescu, EV Ionescu

**Affiliations:** * Techirghiol Balnear and Recovery Sanatorium, Techirghiol, Constanța

The general definition of depression refers to it as a crash of basal mood, with the updating of unpleasant, sad, and threatening affairs. Strong affective participation, deep-seated living, behavioral consensual training are arguments to consider depression a negative hypermeter. As defined above, depression refers to “depression-symptom”, and the definition is only scholastic, because in practice, it appears as a syndrome or nosological entity.

The article refers to this second aspect (syndrome), which was defined, as other authors did, as operational, its components being the ideation, behavioral and somatic resonance of affective disorder. The defining components of depressive syndrome are the following: depressive mood, slowing of thought processes and psychomotor reduction, plus a series of auxiliary symptoms of somatic expression. The depressive disposition is perceived as “vital sadness”, loss of feeli ngs, emptying and inner restlessness, the perceptual gray content.

The case study that we presented involved a 24-year-old patient from Constanta, an automotive professional. In February 2017, he suffered a ski accident by knocking down a tree, which led to a bony polyphase. The reason for the psychological intervention was that the patient was afraid to put the left lower limb in contact with the ground. The psychological evaluation showed that the patient went through a major depression episode. By psychological counseling techniques in addition to recovery therapy, the patient’s evolution improved, and he managed to move normally on long distances.

# Meeting report: INJECTED CORTICOSTEROIDS IN TREATING PLANTAR HEEL PAIN

**Authors:** AM Mirita, N Ghiulcin

Introduction: Plantar heel pain is typically noticed when a person takes the first steps after being inactive or after weight bearing. The pain may get better by itself without treatment. However, it can persist for months and be incapacitating. Treatments include painkillers, heel and arch supports, exercises, shock wave therapy, and local steroid injections.

We assessed the effects of injected steroids to treat adults with painful soles of heels (plantar heel pain) compared to fake treatment (placebo - injections of salt water) or no treatment.

Material and methods: We included 39 studies that involved a total of 2492 adults. The average ages of the participants in the studies ranged from 34 years to 59 years. When reported, most participants had heel pain for several months.

Studies were usually conducted in outpatient specialty clinics of hospitals in 17 countries. Steroid injections were usually given with a local anaesthetic agent. Study follow-up was from one month to over two years.

The studies compared steroid injection with placebo or no treatment (8 studies); tibial nerve block with anaesthetic (2 studies); heel pads (4 studies); oral anti-inflammatory drugs (NSAIDs) (2 studies); an intensive exercise programme (1 study); shock wave therapy (5 studies); laser (2 studies); radiation therapy (1 study); local NSAID injection (1 study); platelet-rich plasma injections (5 studies); 87 injection of the person’s own (autologous) blood (2 studies); botulinum toxin (Botox) injections (2 studies); frozen (cryopreserved) human amniotic membrane injection (1 study); localized peppering involving multiple pricking of the tissues by using an inserted needle (1 study); dry needling (1 study); and mini scalpel-needle release (1 study). We also compared different techniques of local steroid injection (5 studies).

Results: The eight studies comparing steroid injection with placebo or no steroid injection control provided evidence on heel pain, function, serious adverse events, and treatment failure. No studies reported on time to return to work or other activities or short-term adverse events, such as injection-site pain. Steroid injection may slightly reduce heel pain for up to one month after treatment, but not on the longer term including up to six months. We are very unsure whether steroid injection affects longer-term function or reduces treatment failure. There were no serious adverse events, such as infection, reported by these studies. However, these are known to be rare events and we looked at the evidence in all the studies in the review. Of the 21 studies that reported adverse events, two studies reported three infections and two ruptures of heel tissues in relation to steroid injections.

Conclusions: There is low quality evidence that local steroid injections may slightly reduce heel pain up to one month but not subsequently. Although serious complications relating to steroid injection were rare, these were underreported in the included studies and more cannot be ruled out.

# Meeting report: RETROSPECTIVE STUDY ABOUT REHABILITATION AFTER LUMBAR DISK SURGERY IN PATIENTS HOSPITALIZED IN 2016 IN TECHIRGHIOL BALNEAR AND RECOVERY SANATORIUM

**Authors:** V Marin, S Demirgian, D Profir, M Anghel, F Sterghiu

Introduction: In 2016, 836 patients with lumbar disk surgery were hospitalized in Techirghiol Balnear and Recovery Sanatorium.

Background: Complex rehabilitation programs (medical and balnear treatment, physical therapy, massage and kinesiotherapy) are available for individuals after lumbar disk surgery in Techirghiol Balnear and Recovery Sanatorium.

Objectives: A large screening of these patients regarding their age, number of lumbar disk surgeries, level of disk surgical intervention, possible cause of disk hernia, symptoms at admission, clinic evaluation, and type of medical and balnear treatment applied in the Sanatorium.

Method: Statistic analyses of all parameters and correlated data.

Results and conclusion: The complex treatment applied in the Sanatorium reduces pain after lumbar disk surgery with a positive impact on the quality of life and disability and decreases the appearance of a new disk hernia. The treatment should be individualized according to the patients and their symptoms. The patient can return faster to work and other daily activities.

# Meeting report: IMPROVEMENT OF QUALITY OF LIFE PARAMETERS IN PATIENTS WITH DEGENERATIVE LOW BACK PAIN SYNDROMES UNDER SPECIFIC TECHIRGHIOL LAKE BALNEAR THERAPY

**Authors:** AA Lupu, I Albadi, EV Ionescu, ZM Bobe, M Cucu, C Ciobotaru, C Oprea, MG Iliescu

**Affiliations:** * ”Ovidius” University, Faculty of Medicine, Constanţa, Romania; ** ”Sf. Andrei” Clinical County Emergency Hospital, Constanţa, Romania; *** Techirghiol Balnear and Recovery Sanatorium, Constanţa, Romania; **** Mirage Medspa Eforie Nord, Constanţa, Romania; ***** Eforie Nord Rehabilitation Hospital, Constanţa, Romania chirghiol Balnear and Recovery Sanatorium, Techirghiol, Constanța

Objectives. Low back pain affects almost 80% of the population over a lifetime. Degenerative pathology of the lumbosacral spine has a defining feature: diversity, which is determined by the great variety of the anatomical structures. The aim of this study was to evaluate the quality of life parameters in patients with low back pain of degenerative cause under specific Techirghiol lake balnear therapy.

Material and Methods. We evaluated 50 patients with low back pain, hospitalized for rehabilitation treatment in Techirghiol Balnear and Recovery Sanatorium, for a period of 2 weeks. The patients were divided into 2 groups: the first group received treatment with warm mud baths and the second group received treatment with cold mud baths. Both groups received electrotherapy, kinesiotherapy, and massage. All patients were evaluated with Visual Analogue scale and Roland Morris questionnaire before and after the balnear treatment.

Results. There was a significant improvement of both scales in both groups, but the warm mud baths were more efficient.

Conclusions. Balneotherapy with warm mud baths improve the quality of life parameters of patients with degenerative low back pain.

# Meeting report: THE EVOLUTION OF MORFOMETRIC CHANGES, INDICATORS OF DEVELOPMENT AND EFFORT AFTER HYDROKINETOTHERAPY IN CHILDREN WITH SCOLIOSIS

**Authors:** MG Iliescu, AA Lupu, MS Marinica, D Oprea, I Albadi, S Albina, EV Ionescu, DM Iliescu

**Affiliations:** * ”Ovidius” University, Faculty of Medicine, Constanţa, Romania; ** Eforie Nord Rehabilitation Hospital, Constanţa, Romania; *** ”Sf. Andrei” Clinical County Emergency Hospital, Constanţa, Romania; **** Techirghiol Balnear and Recovery Sanatorium, Constanţa, Romania

Our study aimed to build scoliosis recovery programs for children, that are attractive, easy to apply and to be watched, with the possibility of being permanently modulated and modeled during their application, depending on the evolution of the cases and especially effective for the recovery of this type of affection.

As it resulted from the proposed theme, our evaluation program was intended mainly for children, predominantly of school age, whose presentation in the recovery programs is higher and whose screening is easier to achieve because of inclusion in a form of education.

The total lot, of 74 subjects, represented the patients selected from the sports medicine and surgery and pediatric orthopedics ambulatory departments of the Clinical Emergency Hospital in Constanta for a period of 2 years.

The statistical study of the groups implied the centralization of the data using the follow-up record, which included the anatomical and radiological parameters in absolute values as well as the somatometric indices, radiological and effort indices.

The comparison of the results obtained by different types of therapy, applied to different types of scoliosis, has allowed us to demonstrate without doubt the effectiveness of aquatherapy/ hydrokinetotherapy. This proved to be much more effective, more attractive, and less costly for this category of patients.

# Meeting report: THE SEA: SOURCE OF FOOD AND HEALTH

**Authors:** T Zaharia, M Golumbeanu, M Nenciu, RE Almășan, EV Ionescu

**Affiliations:** * “Grigore Antipa” National Institute for Marine Research and Development, Constanța, Romania; Balkan Environmental Association (B.EN.A.), Alexander Technological Education Institute of Thessaloniki, Greece; ** Techirghiol Balnear and Recovery Sanatorium, Constanţa, Romania; *** ”Ovidius” University, Faculty of Medicine, Constanţa, Romania

**Abstract**

The paper outlines some considerations on the importance of the Sea for humans, as a source of food and health. The benefits of the Sea for human health and welfare are incommensurable. On the one hand, the nutritional benefits of seafood consumption (rich in proteins and Omega 3 fatty acids) make the Sea an indispensable source of food, yet not an inexhaustible one. On the other hand, marine science has also revealed other additional benefits for human health.

What is our connection with the sea?

- Air we breathe;

- Food from the dish;

- Ingredients from medical treatments;

- Jobs and the economy;

- Resources offered.

**Keywords:** benefits, sea, human health, welfare

# Meeting report: ARTEMIA SALINA (LINNAEUS, 1758) AND ITS DOUBLE IMPORTANCE: LIVE FEED FOR AQUACULTURE AND SAPROPELIC MUD GENERATOR

**Authors:** M Nenciu, A Țoțoiu, G Harcotă, E Bișinicu, DM Roșioru, T Zaharia, V Niță, ER Almășan, EV Ionescu

**Affiliations:** * “Grigore Antipa” National Institute for Marine Research and Development, Constanţa, Romania; ** Techirghiol Balnear and Recovery Sanatorium, Constanţa, Romania; *** ”Ovidius” University, Faculty of Medicine, Constanţa, Romania

Artemia salina (Linnaeus, 1758) is a micro-crustacean, well adapted to the conditions of severely hypersaline environments, such as the ones Lake Techirghiol impose on survival and reproduction. The most obvious survival strategy is a highly efficient osmoregulation system to withstand up to 10 times the salt concentration of ordinary seawater. Under extremely critical environmental conditions, the brine shrimp takes refuge by producing a highly resistant encysted embryo (cyst) capable of severe dehydration, enabling an escape from population extinction. This peculiarity of cyst formation allows this crustacean to be extensively used in aquaculture. Among the live diets used in the larviculture of fish and shellfish, nauplii of the brine shrimp represent the most widely used food item. Annually, over 2,000 metric tons of dry Artemia cysts are marketed worldwide for on-site hatching into 0.4 mm nauplii. Indeed, the unique property of the small branchiopod crustacean Artemia to form dormant cysts, may account largely for the designation of a convenient, suitable, or excellent larval food source that it has been credited with. The cysts are available year-round in large quantities along the shorelines of hypersaline lakes, coastal lagoons and solar saltworks scattered over the five continents. After harvesting and processing, cysts are made available in cans as storable on demand live feed.

Upon some 24-h incubation in seawater, these cysts release free-swimming nauplii that can be directly fed as a nutritious live food source to the larvae of a variety of marine as well as freshwater organisms, which makes them the most convenient, least labor-intensive live food available for aquaculture. In the frame of a controlled feeding experiment on the long-snouted seahorse (Hippocampus guttulatus Cuvier, 1829) developed in our institute, it resulted that brine shrimps are not only the preferred prey by adult seahorses, but also led to a higher increase in length and weight of the studied specimens, due to their high nutritional potential. Moreover, the role of this phyllopod crustacean in peloidogenesis of Lake Techirghiol is well acknowledged. Aretmia salina is of great importance for Lake Techirghiol, as bodies fall to the bottom of the lake and, by bacterial decomposition, they form sapropelic mud with active mineral components that give great therapeutic value. This paper underlines this double role of the crustacean, on the one hand as source of food in aquaculture and, on the other hand, as bio-resource for mud generation.

**Keywords:** brine shrimp, live feed, aquaculture, hypersaline, mud

# Meeting report: THE NATURAL CONDITIONS FOR SAPROPELIC MUD FORMATION IN LAKE TECHIRGHIOL AND ITS CHARACTERISTICS

**Authors:** DM Roşioru, E Bişinicu, O Marin

**Affiliations:** * “Grigore Antipa” National Institute for Marine Research and Development, Constanţa, Romania

Romania is among the European countries with a remarkable balnear potential and the use of natural treatment factors for therapeutic purposes dates back to ancient times. Techirghiol ecosystem in Romania is unique because of its hypersaline character and because of the large productivity in the formation of the sapropelic mud with therapeutic properties.

The composition of peloids (therapeutic muds) is different and depends on the natural conditions of their formation; they are the sediments that form as a result of the decomposition of flora and fauna under the influence of microbiological activity.

The presence of the green macroalgae Chladophora and aquatic arthropod Artemia salina under the physicochemical and geo-hydrological conditions of Lake Techirghiol contribute to mineralization and to the way mud with sapropelic character is formed.

The paper presented the ecological and biological aspects of Cladophora and Artemia salina and the natural conditions for therapeutic mud production in Lake Techirghiol.

Also, the physicochemical and biochemical characteristics of sapropelic mud from Lake Techirghiol and its therapeutic properties were presented.

**Keywords:** Lake Techirghiol, sapropelic mud, natural conditions, Cladophora, Artemia salina, characteristics
